# Experimental Study on the Dynamic Properties of Granite with Filled Joints of Different Thicknesses

**DOI:** 10.3390/ma18050936

**Published:** 2025-02-21

**Authors:** Zhide Wang, Jiaxing An, Yuanyou Xia, Yingying Si

**Affiliations:** 1 School of Civil Engineering and Architecture, Wuhan University of Technology, Wuhan 430070, China; 348053@whut.edu.cn (J.A.); xiayy1965@whut.edu.cn (Y.X.); 2 Hubei Communication Investment Construction Group Co., Traffic Engineering Company, Wuhan 430070, China; siyingying@whut.edu.cn

**Keywords:** filling joints, dynamic characteristics, energy dissipation characteristics, the law of destruction, Hopkinson compression rod

## Abstract

To investigate the dynamic characteristics, energy dissipation patterns, and failure modes of granite with filled joints of varying thicknesses under impact loading, we utilized the Split Hopkinson Pressure Bar (SHPB) test setup for impact tests on both unfilled and filled granite samples. Additionally, a high-speed camera was used to capture the dynamic failure and crack propagation processes of the rock samples in real time. The results indicate that the thickness of the filling material significantly affects the stress–strain behavior of jointed rock masses, particularly in terms of characteristics of stress variation and post-peak morphology. In comparison to unfilled jointed rock samples, a distinct “stress bimodal” phenomenon is present, and the rebound of strain following the peak gradually decreases. The fracture patterns observed in the jointed rock samples are primarily characterized by tensile failure. Damage is notably more pronounced on the left side of the samples (near the incident bar), the lower side, and in the areas filled with gypsum. The most severe degree of damage occurs when the filling thickness is 7.56 mm. As the thickness of the filling increases, the dynamic compressive strength of the rock mass diminishes, and the peak strain first increases and then decreases. Concurrently, the energy reflection coefficient of the rock mass increases linearly, while the energy transmission coefficient declines linearly. Furthermore, the energy dissipation ratio first increases and then decreases. The test data reveal that the critical filling thickness influencing the dynamic properties, energy absorption characteristics, and damage degree of jointed rock samples falls within 4.91 mm to 7.56 mm.

## 1. Introduction

Complex geological structural movements and human engineering activities have formed numerous joints and fractures in natural rock masses, with most joints containing fillings of a certain thickness. In light of this, jointed rock masses are categorized into filled and unfilled types. Compared to intact rock masses, the deformation and failure characteristics of jointed rock masses are more complex, particularly as the presence of fillings can alter the rock mass’s joint strength and stability [1,2]. Recently, the rapid development of socio-economic conditions and technology has led to the expansion of rock engineering into deeper rock formations in the fields of mining, energy, transportation, and hydropower, both domestically and internationally. However, in engineering construction, the shock waves generated by blasting excavations can cause deformation or even damage to filled jointed rock masses, which severely affects engineering safety [3,4,5].

Researchers around the world have extensively studied the dynamic properties of rocks, resulting in numerous beneficial findings. Research on SHPB impact tests for intact rocks has become quite advanced. For example, Mishra S. et al. [6] utilized an SHPB apparatus to test five Himalayan phyllite specimens of varying dimensions. The results indicated that the size of the phyllite specimens, the magnitude of the air gun pressure, and the impact bar’s length significantly influence the specimens’ dynamic characteristics. Liu et al. [7] examined the stress–strain curves of phosphate ore at various strain rates and found that the dynamic elastic modulus is not significantly affected by the strain rate. Mishra S. [8] et al. investigated the dynamic properties of dense basalt, altered basalt, and granite under high strain rates. Additionally, they proposed two correlation equations for the dynamic strength enhancement factor of rocks. Hou et al. [9] performed constant strain rate SHPB impact tests on granite and introduced a novel strength criterion to elucidate the relationship between the Dynamic Increase Factor (DIF) and strain rate. By employing the Zhu–Wang–Tang viscoelastic model, they formulated a constitutive model that accurately characterizes the dynamic mechanical properties of granite. A variety of research studies have investigated the dynamic properties of non-filled jointed rock masses. For instance, Kumar et al. [10] examined the mechanical behavior of heat-treated rock specimens with defects under dynamic loading conditions, both in the ungrouted and grouted states. The results revealed that the presence of grouting significantly enhanced the mechanical properties of the specimens. Li et al. [11] conducted dynamic impact tests on rectangular marble specimens that contained joints to examine how the joint dip angle, geometry, and number affect the dynamic mechanical properties, fracturing behavior, and energy evolution characteristics of the rock. They employed a high-speed camera to monitor and record the processes of crack initiation, coalescence, and failure in the specimens in real time. G. Swoboda et al. [12] developed an anisotropic and nonlinear damage model for jointed rock masses to describe their mechanical behavior, which was subsequently applied to the mechanical analysis of tunnels. Li et al. [13] used an improved SHPB apparatus to analyze the effects of joint roughness on the seismic quality factor of rock masses and the attenuation of stress wave energy within the rock masses. Li et al. [14] conducted impact tests on red sandstone specimens with different joint angles to study the stress wave propagation and fracture evolution patterns in specimens composed of two rock blocks. Yan et al. [15] conducted experiments and found that rock joints exhibit viscous characteristics under dynamic loads. They proposed the Kelvin–Voigt model and validated it, suggesting that the viscous characteristics of joints are related to their contact area and roughness. Filled joints can have a significant impact on the dynamic properties of rock masses, as demonstrated in various studies. For example, Kumar et al. [16] conducted SHPB apparatus tests on rock masses with intact joints, unfilled joints, cement-filled joints, and epoxy resin-filled joints. High-speed cameras and digital imaging techniques were employed for observation. The study established the patterns of how the dynamic characteristics of the specimens varied with strain rate. Chai et al. [17] carried out static and dynamic compression tests to evaluate the mechanical properties of prefabricated artificially filled rock joint specimens, which varied in filling materials and joint thicknesses. They analyzed the strength, deformation, wave propagation, and energy dissipation patterns of these filled jointed rock masses. Li et al. [18] conducted an experimental study using the SHPB apparatus to investigate the propagation of stress waves in filled joint granite. Their research revealed that both the joint width and water content significantly influence the dynamic stress–strain relationship of grouted jointed rock. Han et al. [19] used an improved SHPB system to conduct experimental studies on sandwich sandstone specimens filled with cement mortar of different thicknesses. They analyzed the impact of filled joints on the dynamic response of rock specimens. Mahdi et al. [20] utilized the discrete element method to analyze the failure mechanism of rock with filled joints. Additionally, they conducted experiments on the shear behavior of rock with filled joints under different normal loads. These experiments were carried out to validate the effectiveness of the proposed viscous model in characterizing the shear properties of rock with filled joints. Chai et al. [21] conducted a series of impact tests on artificially filled jointed rock specimens to evaluate their dynamic mechanical properties and the evolution of cumulative damage under repeated impacts.

In summary, there is a wealth of research on the dynamic characteristics of intact rocks and unfilled jointed rock masses. However, there remains considerable scope for further research on the dynamic properties and failure mechanisms of filled jointed rock masses. To investigate the impact of filling thickness on the dynamic properties of jointed rock masses, granite with joints was chosen as the subject of the study. The research examined both unfilled and filled joint types, employing SHPB dynamic impact tests to assess their dynamic mechanical properties and patterns of energy dissipation. A high-speed camera recorded the experimental process in real-time, allowing for the observation of dynamic failure modes and crack propagation in the samples. This research provides a theoretical foundation for evaluating stability and ensuring safety in blasting projects in tunnels, slopes, and mine roadways.

## 2. The Experimental Design

### 2.1. The Experimental Setup

**Figure 1:**
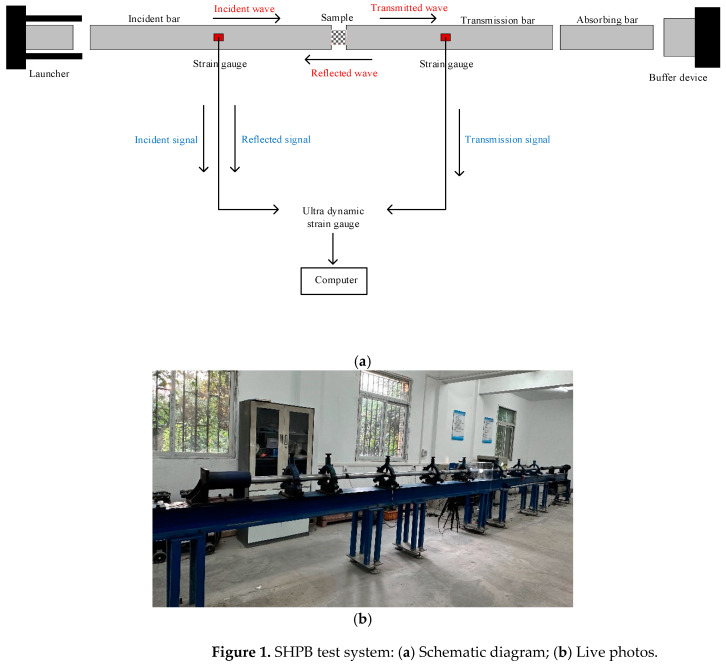
SHPB test system: (**a**) Schematic diagram; (**b**) Live photos.

The SHPB experimental device produced by Xi'an Baina Electronic Technology Co., Ltd. in China was used to carry out dynamic impact test on jointed rock samples. This apparatus primarily consists of a launching system, a pressure bar system, a buffering device, and a data acquisition system. The pressure bar system includes an impact bar, an incident bar, a transmission bar, and an absorbing bar, all made from the same high-strength steel material with a diameter of 60 mm, an elastic modulus of 210 GPa, and a longitudinal wave velocity of 5000 m/s. The lengths of both the incident and transmission bars are 2.8 m. A schematic and a field photograph of the SHPB experimental setup are presented in Figure 1.

### 2.2. Sample Preparation

**Figure 2:**
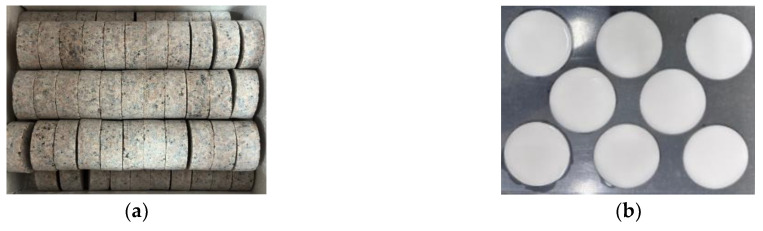
Sample: (**a**) Granite sample; (**b**) Gypsum sample.

Due to the challenges in obtaining naturally filled jointed rock masses, this study employs artificial jointed rock samples made from a combination of granite and rock-like materials. The granite material used in the experiments was sourced from the same quarry and is characterized by good integrity and uniformity. To ensure that the aspect ratio of the fabricated samples is close to 1:1 [22], the granite was machined into cylindrical samples with a diameter of 50 mm and a height of 22 mm. The rock-like material selected was plaster, which was fashioned into plaster fillings of various thicknesses with a diameter of 50 mm, as shown in Figure 2. The physical and mechanical parameters of the rock and filling materials are presented in Table 1.

(1) Unfilled Jointed Rock Samples: Two cylindrical granite samples were taken, and a thin layer of plaster was applied between the two rock blocks. The two blocks were then bonded together and secured with rubber bands for 48 h.

(2) Filled Jointed Rock Samples: Plaster was used to bond two rock blocks and plaster-filling pieces together, and they were fixed for 48 hours to create five kinds of jointed rock samples of different thicknesses.

**Figure 3:**
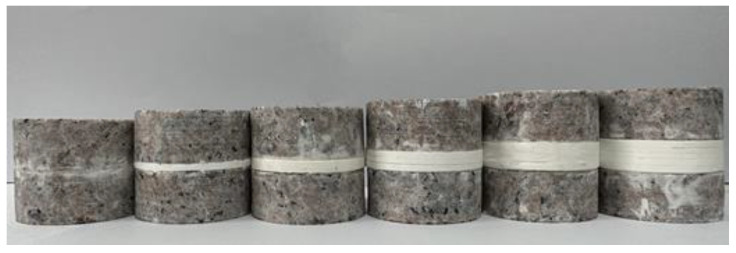
Jointed rock samples.

Each type of jointed rock sample was produced in triplicate to ensure the consistency required for parallel experiments. In accordance with the fundamental requirements of the “Rock Dynamic Properties Testing Code” [22], it was ensured that both end surfaces were smooth, with parallelism and perpendicularity controlled within the range of 0.01 to 0.02 mm. Considering the impact of errors during the experimental process and to ensure the credibility of the experimental data, each type of rock sample underwent three replicate tests. The prepared jointed rock samples are shown in Figure 3, and the physical parameters of different types of rock samples are presented in Table 2.

### 2.3. Experimental Principle

The experiment adheres to the assumptions of one-dimensional planar elastic wave propagation and stress uniformity, utilizing the three-wave method formula for data processing. This involves collecting incident strain, *ε_i_* (*t*), reflected strain, *ε_r_* (*t*), and transmitted strain, *ε_t_* (*t*), using two sets of strain gauges affixed to the incident and transmitted bars. Additionally, based on the assumption of uniform stress distribution: *ε_i_* + *ε_r_* = *ε_t_*, the formulas are simplified to calculate the stress, *σ* (*t*), strain, *ε* (*t*), and strain rate, *έ*(*t*), of the sample as follows:

(1) $$\sigma \left(t\right)=\frac{A_{0}}{A_{s}}E_{0}\varepsilon_{t}\left(t\right)$$

(2) $$\varepsilon \left(t\right)=-\frac{2C_{0}}{L_{s}}\int_{0}^{t}\varepsilon_{r}\left(t\right)\mathrm{dt}$$

(3) $$\overset{\prime }{\varepsilon }\left(t\right)=-\frac{2C_{0}}{L_{s}}\varepsilon_{r}\left(t\right)$$

In the equations: *t* represents the duration of the stress wave (s), *E*_0_ is the elastic modulus of the pressure bar (GPa), *A*_0_ is the cross-sectional area of the pressure bar (mm^2^), *A_s_* is the initial cross-sectional area of the sample (mm^2^), *C*_0_ is the longitudinal wave velocity of the pressure bar (m/s), and *L_s_* is the initial length of the sample (mm).

## 3. The SHPB Testing Process for Filled Jointed Granite

### 3.1. Dynamic Stress Equilibrium Verification

After several preliminary experiments, it was determined that the impact load for this SHPB test should be set at 0.08 MPa. At this load level, the dynamic failure and crack propagation processes of the jointed granite can be clearly observed using high-speed photography.

**Figure 4:**
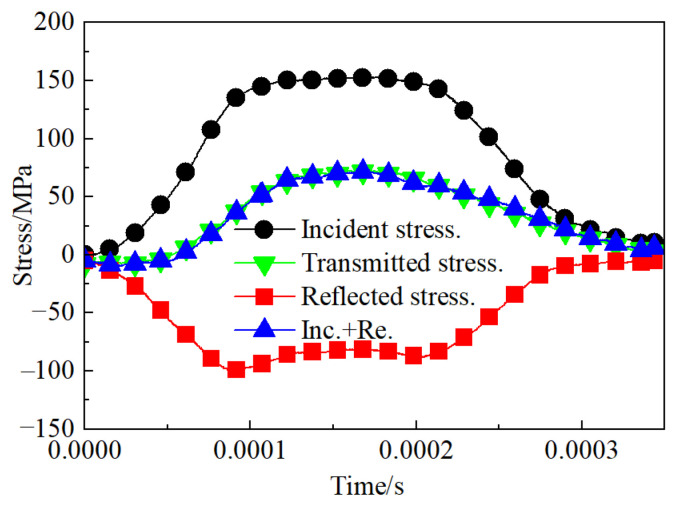
Dynamic stress balance diagram.

Based on the one-dimensional stress wave theory, during the SHPB impact test, the stress at both ends of the rock sample must reach an equilibrium state before failure. Figure 4 illustrates the stress equilibrium at both ends of the jointed rock specimen. The reflected stress propagates in the opposite direction to the incident stress; hence its signal value is negative. Consequently, the stress at the incident end (the sum of the incident stress and the reflected stress) is essentially consistent with the transmitted stress. The stresses at both ends of the rock sample satisfy the equilibrium condition, namely *σ*_1_(*t*) = *σ*_2_(*t*), indicating that the sample is in a state of dynamic stress equilibrium during the impact test, ensuring the validity of the experimental data.

### 3.2. The Dynamic Failure and Crack Propagation Process of Jointed Granite

**Figure 5:**
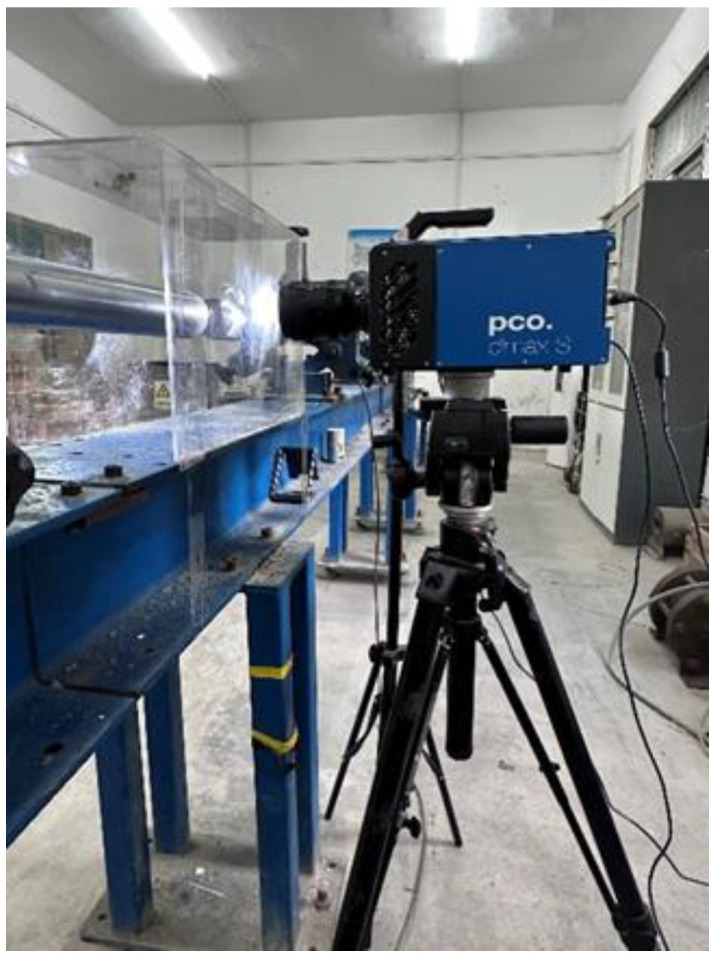
High-speed camera.

In order to facilitate the observation of the dynamic failure and crack propagation process of jointed rock samples under impact load, the experiment was captured in real time by the pco.dimax S4 digital high-speed camera produced by PCO AG, as shown in Figure 5. The camera has a resolution of 4 megapixels (2016 × 2016) and a frame rate of 1102 frames per second (color), enabling it to capture images at extremely high speeds suitable for various applications requiring the capture of high-speed motion and transient processes. Moreover, its light sensitivity and dynamic range ensure high-quality image capture even under low-light conditions, making it widely used in the field of material impact research and other related areas.

**Figure 6:**
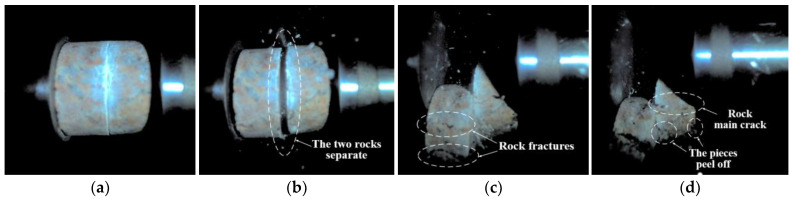
Dynamic failure process of unfilled jointed rock sample: (**a**) Preimpact; (**b**) 8.167 ms; (**c**) 58.076 ms; (**d**) 77.132 ms.

**Figure 7:**
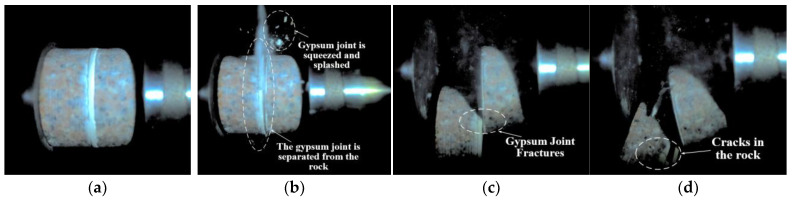
Dynamic failure process of a 2.40 mm filled jointed rock sample: (**a**) Preimpact; (**b**) 5.445 ms; (**c**) 49.002 ms; (**d**) 59.891 ms.

**Figure 8:**
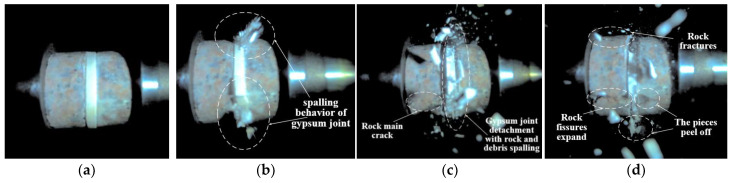
Dynamic failure process of a 4.91 mm filled jointed rock sample: (**a**) Preimpact; (**b**) 0.907 ms; (**c**) 9.982 ms; (**d**) 66.243 ms.

**Figure 9:**
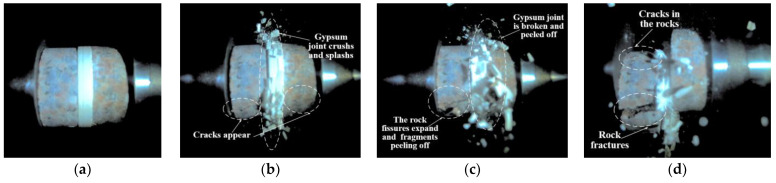
Dynamic failure process of 7.56 mm filled jointed rock sample: (**a**) Preimpact; (**b**) 6.352 ms; (**c**) 19.056 ms; (**d**) 81.669 ms.

**Figure 10:**
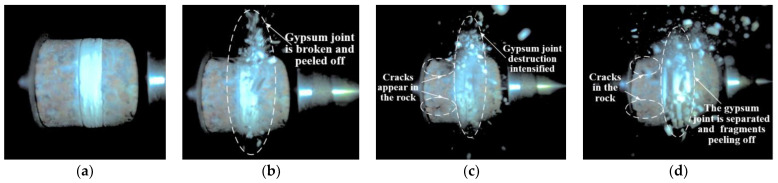
Dynamic failure process of 10.73 mm filled jointed rock sample: (**a**) Preimpact; (**b**) 5.445 ms; (**c**) 21.779 ms; (**d**) 43.557 ms.

**Figure 11:**
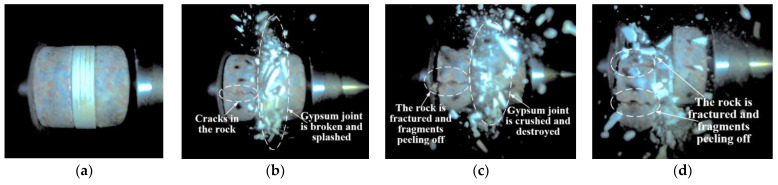
Dynamic failure process of 12.15 mm filled jointed rock sample: (**a**) Preimpact; (**b**) 4.537 ms; (**c**) 14.519 ms; (**d**) 79.855 ms.

Figure 6 illustrates the dynamic failure process of unfilled jointed rock samples, while Figure 7, Figure 8, Figure 9, Figure 10 and Figure 11 show the crack propagation processes in filled jointed rock samples of different thicknesses under dynamic impact.

As shown in Figure 6, at 8.167 ms, the unfilled jointed granite is impacted, and the stress wave is transmitted from the incident bar to the struck end of the sample (the left end of the photograph), causing the two rock blocks to separate. At this moment, a portion of the stress wave is transmitted to the joint, where it reflects to the left side of the rock mass due to the wave impedance effect. By 58.076 ms, significant tensile cracks developed in the rock mass on the left side of the joint and extended to rock fracture. Meanwhile, another portion of the stress wave continues to propagate in the original direction towards the right side of the jointed rock, resulting in tensile cracks and the detachment of rock fragments (at 77.132 ms).

As shown in Figure 7, the jointed rock sample with a plaster filling thickness of 2.40 mm is more susceptible to failure than the unfilled jointed rock samples due to the lower compressive strength of the plaster compared to the rock matrix. When the impact wave reaches the sample, it is primarily divided into three components. A portion of the impact wave is absorbed by the plaster filling, which leads to the failure of the plaster joint first. At 5.445 ms, a crushing separation phenomenon occurs at the edges of the joint, resulting in the pulverization and ejection of the plaster joint. By 49.002 ms, a noticeable fracture is evident in the plaster joint. Another portion of the stress wave reflects off the rock mass on the left side of the joint, and at 59.891 ms, cracks develop in the left rock mass due to tensile stress. In contrast to the unfilled jointed rock samples, the absorption of part of the stress wave by the plaster filling means that the transmitted stress wave is insufficient to cause damage to the rock mass on the right side of the joint. Consequently, no significant cracks are observed in the right rock mass.

As shown in Figure 8, when the impact wave reaches the jointed rock sample with a filling thickness of 4.91 mm, a portion of it is absorbed by the plaster filling. At 0.907 ms, there is a phenomenon of fragment detachment and splashing on both the lower and upper sides of the plaster joint. By 9.982 ms, the joint separates from the rock. A portion of the stress wave, after passing through the joint, is reflected, causing the rock mass on the left side of the joint to develop tensile cracks that continue to expand until the rock fractures. Additionally, another part of the stress wave propagates forward to the rock on the right side of the joint, and at 66.243 ms, the right rock also exhibits cracks accompanied by the detachment of rock fragments.

As the thickness of the plaster filling increases, the capacity of the filling to absorb stress waves reaches its upper limit, the damage degree of the plaster joint is obvious, and the overall damage degree of the sample is intensified. As depicted in Figure 9, with a filling thickness of 7.56 mm, the plaster joint experiences significant pulverization failure at 6.352 ms. During this time, tensile cracks emerge in the lower portions of the rock masses flanking the joint. The lower crack in the left rock mass extends until the rock ultimately fractures at 19.056 ms. By 81.669 ms, additional cracks begin to form in the upper section of the left rock mass, accompanied by the detachment of rock fragments. The reflection of stress waves at the joint leads to the left side of the rock mass being impacted by stress waves twice, resulting in more severe damage on that side. The lower section of the rock mass is particularly vulnerable to failure compared to the upper section. Furthermore, the thickness of the joint filling does not completely prevent stress waves from penetrating into the rock mass on the right side of the joint. Consequently, the right rock mass also shows significant cracks and damage (at 81.669 ms).

As shown in Figure 10, when the filling thickness is 10.73 mm, under the impact, the plaster joint undergoes pulverization failure and separates from the rock matrix. As the thickness of the filling increases, the impedance of the plaster joint to stress waves intensifies, leading to a more significant reflection of these waves. At 5.445 ms, tensile cracks start to develop in the rock mass on the left side of the joint, continuing to propagate until the rock ultimately fractures at 21.779 ms. Fragmentation of the rock is observed at 43.557 ms. Due to the thicker filled joints, most of the stress waves are absorbed and reflected, resulting in significant attenuation of the transmitted stress waves, and no significant cracks are observed in the rock on the right side of the joint.

As shown in Figure 11, at the moment of impact, the jointed rock sample with a filling thickness of 12.15 mm experiences destruction after the plaster joint absorbs the stress waves, resulting in pulverization and splashing phenomena. With the increase in filling thickness, the reflected stress waves intensify. At 4.537 ms, a tensile crack appears in the middle part of the rock mass on the left side of the joint and continues to propagate until 14.519 ms, followed by rock fracture and fragment spalling at 79.855 ms. Due to the increased thickness of the plaster filling, most of the stress waves are reflected, and the stress waves that pass through the plaster joint are insufficient to cause damage to the rock mass on the right side of the joint, with no significant cracks observed.

Overall, the fracture mode of the jointed rock samples is predominantly tensile failure, with the rock mass on the left side of the joint (closer to the incident bar), the lower part of the rock mass, and the plaster filling section being more susceptible to damage. The jointed rock sample with a filling thickness of 7.56 mm exhibited the most severe degree of damage, with evident pulverization failure observed on both sides of the joint and within the plaster filling. The jointed rock sample with a filling thickness of 2.40 mm exhibited the least degree of damage, with only the plaster filling experiencing significant destruction. Cracks were observed in the rock mass on the left side of the joint without overall failure, while no apparent cracks were detected in the rock mass on the right side of the joint.

## 4. Experimental Results and Discussion

### 4.1. Stress–Strain Curve Characteristics

**Figure 12:**
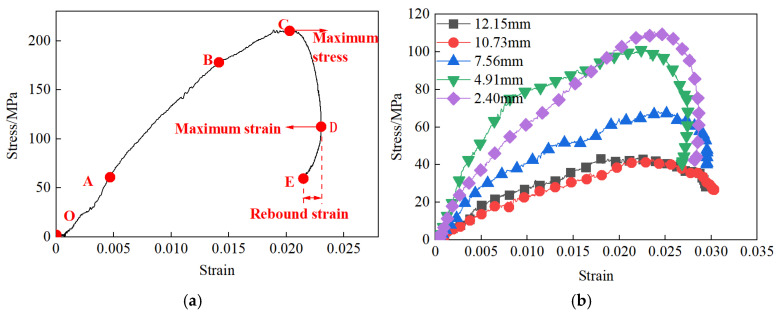
Stress–strain curves of jointed rock samples: (**a**) Unfilled jointed rock sample; (**b**) Filling type jointed rock sample.

The impact test data from unfilled jointed rock samples and five jointed rock samples with varying filling thicknesses were processed using the three-wave method. Representative stress–strain curves were selected to analyze the dynamic mechanical properties of the samples. The filling thicknesses were 2.40 mm, 4.91 mm, 7.56 mm, 10.73 mm, and 12.15 mm, respectively. The stress–strain curves for the rock samples with different joint types are presented in Figure 12.

The stress–strain curves of the rock samples are depicted in Figure 12a. For the unfilled jointed rock mass, the curve can be divided into four phases: (1) Initial compaction phase (segment OA). This phase indicates the closure of initial micro-fractures within the rock sample under compression, representing non-elastic deformation; (2) Linear elastic phase (segment AB). The curve exhibits an approximately linear increase in this phase, with the slope of the curve being considered equivalent to the dynamic elastic modulus of the rock sample; (3) Plastic deformation phase (segment BC). The stress–strain relationship no longer exhibits a linear behavior, with the slope of the curve gradually decreasing. Despite this, stress continues to increase until it reaches the peak stress; (4) Post-peak unloading phase (after point C). This phase can be further divided into two parts: the first unloading stage (segment CD), where stress continuously decreases and the dynamic stress–strain curve rapidly descends, with the slope of the unloading curve being greater than that of the loading curve, indicating that the unloading modulus is greater than the loading modulus; the second unloading stage (segment DE), elastic deformation energy continues to accumulate as cracks propagate in the rock sample, resulting in a decreasing trend in strain. When the rock sample fails, the stored elastic deformation energy is gradually released, creating a phenomenon known as “strain rebound”. This indicates that the rock sample retains some load-bearing capacity even during the post-peak unloading phase.

Compared to the unfilled jointed rock samples, the stress–strain curve patterns of the filled jointed rock samples exhibit significant differences, primarily characterized by varying degrees of ‘stress bimodal’ and post-peak morphologies, as shown in Figure 12b. The reason for the differences in the stress–strain curve patterns between filled and unfilled jointed rock samples is that the gypsum filling has a lower compressive strength compared to the rock matrix. Upon impact, the gypsum filling fails first, manifesting as the first stress peak on the stress–strain curve. As the rock matrix continues to be compressed until the rock sample undergoes overall failure, the second stress peak is reached. When the filling thickness is 4.91 mm, the “stress bimodal” phenomenon in the jointed rock samples is the most pronounced. As the filling thickness increases, the degree of “strain rebound” continuously decreases, and the load-bearing capacity of the rock samples during the post-peak unloading phase is reduced. When the filling thickness is 10.73 mm and 12.15 mm, the jointed rock samples exhibit almost no “strain rebound” phenomenon, indicating that the jointed rock samples have almost no load-bearing capacity after failure.

### 4.2. The Relationship Between Dynamic Properties and Filling Thickness

**Figure 13:**
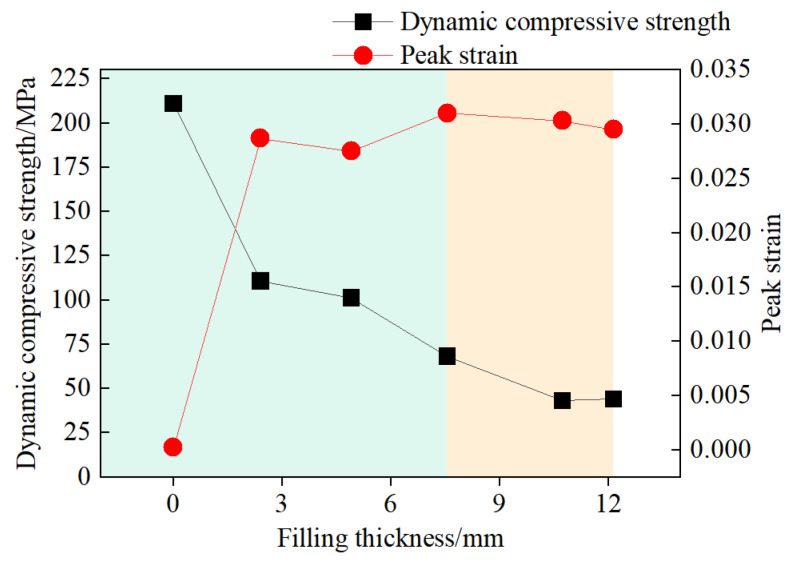
The relationship between dynamic compressive strength and filling thickness.

In dynamic impact tests, the primary dynamic properties of rock samples include peak stress and peak strain. The peak stress, also referred to as the dynamic uniaxial compressive strength of rock [23,24], represents the ultimate stress value that the rock can withstand during the impact process; the peak strain reflects the maximum deformation that the rock can endure under dynamic impact loading [25]. The relationship between the dynamic compressive strength and peak strain of jointed rock samples and the filling thickness is depicted in Figure 13.

The dynamic compressive strength of unfilled jointed rock samples is 211.05 MPa. In contrast, the dynamic compressive strengths of jointed rock samples with filling thicknesses of 2.40 mm, 4.91 mm, 7.56 mm, 10.73 mm, and 12.15 mm are 110.76 MPa, 101.22 MPa, 68.29 MPa, 43.15 MPa, and 44.21 MPa, respectively. The dynamic compressive strength decreases with an increase in filling thickness. Additionally, as the filling thickness grows, the likelihood of internal fractures developing within the filling material under dynamic loading also increases. This results in a decreasing overall ability of the jointed rock samples to withstand impact loads.

The peak strain of unfilled jointed rock samples is 2.3 × 10^−2^, while the peak strains of jointed rock samples with filling thicknesses of 2.40, 4.91, 7.56, 10.73, and 12.15 mm are 2.87 × 10^−2^, 2.75 × 10^−2^, 3.10 × 10^−2^, 3.03 × 10^−2^, and 2.95 × 10^−2^, respectively. Figure 13 reveals the pattern of how the peak strain of the samples varies with filling thickness. Under the same impact conditions, the peak strain of filled jointed rock samples is consistently higher than that of unfilled jointed rock samples. Additionally, the peak strain tends to increase initially with greater filling thickness, but then it starts to decrease as the thickness continues to rise. As the filling thickness increases from 0 mm to 7.56 mm, the overall ability of the samples to resist deformation is reduced due to the lower compressive strength of the gypsum compared to the rock matrix, resulting in an increase in peak strain. When the filling thickness increases from 7.56 mm to 12.15 mm, the filling thickness plays a certain cushioning role, leading to a slight decrease in peak strain, with a critical joint thickness (approximately 7.56 mm) at which the peak strain undergoes a significant change. The sudden change in peak strain can be seen from the change in background color in the Figure 13.

## 5. Patterns of Energy Evolution

### 5.1. Principle of Energy Dissipation in Jointed Rock Mass

In SHPB impact testing, when the impact bar strikes the incident bar at a certain velocity, energy is generated in the form of stress waves, which include incident energy *W_i_*, reflected energy *W_r_* and transmitted energy *W_t_*. Furthermore, a minor fraction of the energy is dissipated, which includes the energy *W_s_* absorbed by the damage and destruction of the jointed rock samples, the kinetic energy *W_sk_* generated by the ejection of fragmented rocks during impact, and other forms of dissipated energy such as thermal energy.

According to the research of previous investigators, *W_sk_* constitutes 5–10% of W*_s_*, which is a minor proportion and, therefore, is chosen to be disregarded in this study [26]. The experiments were carried out in a controlled indoor environment with a constant temperature, ensuring that thermal exchange with the outside environment was negligible. As a result, thermal energy can be effectively disregarded. Other forms of dissipated energy were minimal and are also considered negligible. Therefore, the absorbed energy *W_s_* can be approximated as the total dissipated energy, which can be calculated using the following formula:

(4) $$W_{s}=W_{i}-W_{r}-W_{t}$$

Utilizing the stress wave data obtained from the SHPB (Split Hopkinson Pressure Bar) test, by the law of conservation of energy and one-dimensional stress wave theory, the incident energy, *W_i_*, reflected energy, and transmitted energy, *W_t_*, can be calculated using the following formulas, respectively [13]:

(5) $$W_{i}=\frac{A_{0}C_{0}}{E_{0}}\int_{0}^{t}\sigma_{i}^{2}\left(t\right)\mathrm{dt}=E_{0}A_{0}C_{0}\int_{0}^{t}\varepsilon_{i}^{2}\left(t\right)\mathrm{dt}$$

(6) $$W_{r}=\frac{A_{0}C_{0}}{E_{0}}\int_{0}^{t}\sigma_{r}^{2}\left(t\right)\mathrm{dt}=E_{0}A_{0}C_{0}\int_{0}^{t}\varepsilon_{r}^{2}\left(t\right)\mathrm{dt}$$

(7) $$W_{t}=\frac{A_{0}C_{0}}{E_{0}}\int_{0}^{t}\sigma_{t}^{2}\left(t\right)\mathrm{dt}=E_{0}A_{0}C_{0}\int_{0}^{t}\varepsilon_{t}^{2}\left(t\right)\mathrm{dt}$$

### 5.2. Energy–Time Curve

**Figure 14:**
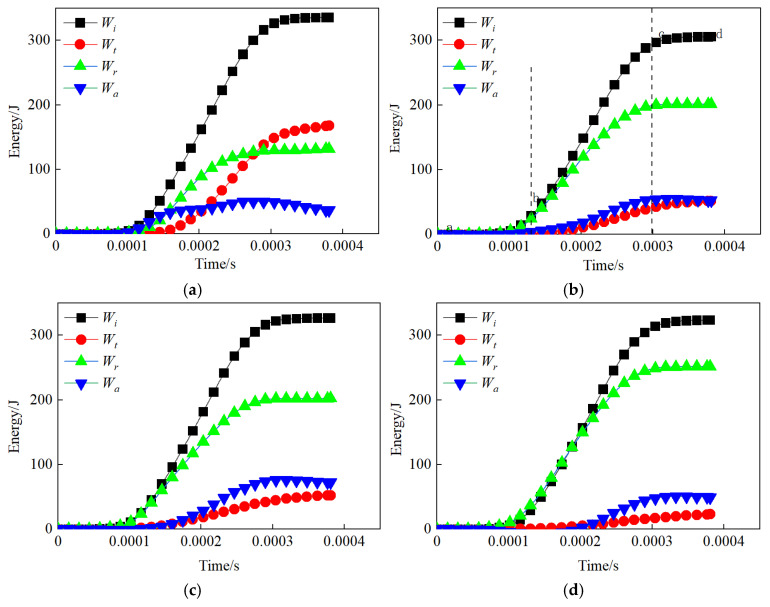

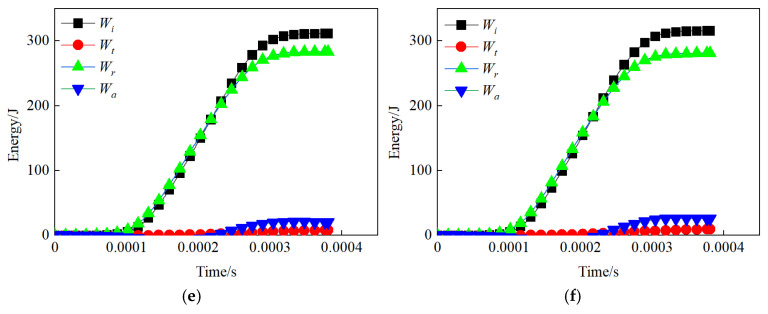
Energy time history curve: (**a**) Unfilled; (**b**) 2.40 mm; (**c**) 4.91 mm; (**d**) 7.56 mm; (**e**) 10.73 mm; (**f**) 12.15 mm.

Figure 14 presents the energy-time curves for unfilled jointed rock samples and five jointed rock samples with different filling thicknesses. The energy evolution process can generally be divided into three stages, with the example of a jointed rock sample with a filling thickness of 2.40 mm used for illustration, as shown in Figure 14b:

(1) The compaction phase (ab segment) is where all energy components gradually increase. The absorbed energy is primarily used for compacting filled joints and rock mass fractures, which corresponds to the initial compaction phase of the stress–strain curve. (2) The energy stable growth phase (segment bc) is characterized by constant slopes in the energy-time curves, with absorbed energy increasing rapidly and steadily. During this phase, the overall fractures in the sample expand stably, and both strain and stress continue to rise until peak strain and dynamic compressive strength are reached. This corresponds to the linear elastic phase and the plastic phase of the stress–strain curve. (3) The failure phase (segment cd) is where the slopes of the energy-time curves become zero, indicating that all energy components have reached their peak values and are no longer developing. At this point, the sample is completely destroyed, absorbing almost no additional energy.

When comparing unfilled jointed rock samples to filled jointed rock samples, we find a similar trend in incident energy. The reflected energy increases as the filling thickness rises, while the transmitted energy steadily decreases with increasing filling thickness. Meanwhile, the absorbed energy initially increases before decreasing as the filling thickness grows. By examining the relationships between various filling thicknesses and the incident energy, reflected energy, transmitted energy, and absorbed energy in joint granite, we can understand the propagation behavior of stress waves. It is evident that as filling thickness increases, the impedance effect becomes more significant, resulting in higher reflected energy, a gradual decline in transmitted energy, and absorbed energy that first rises and then falls.

### 5.3. The Impact of Filling Thickness on Energy Dissipation

The energy reflection coefficient, energy transmission coefficient, and energy dissipation ratio are introduced to gain a more intuitive understanding of the relationship between reflected energy, transmitted energy, dissipated energy, and incident energy. These parameters facilitate the analysis of the impact of filling thickness on energy dissipation in jointed rock masses [26].


(8) $$\eta_{s}=\frac{W_{r}}{W_{i}}\cdot \eta_{s}=\frac{W_{t}}{W_{i}}\cdot \eta_{s}=\frac{W_{s}}{W_{i}}$$

#### 5.3.1. The Relationship Between Energy Coefficients and Filling Thickness

**Figure 15:**
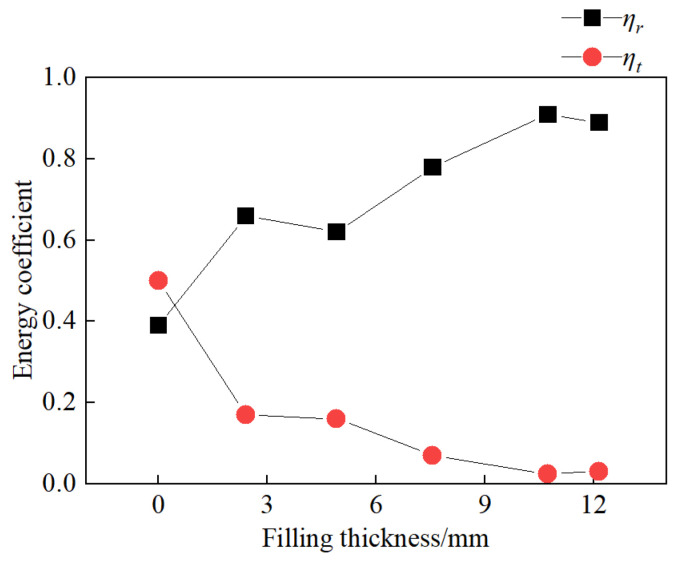
The relationship between energy coefficient and filling thickness.

The relationship between the energy reflection coefficient, *ƞ_r_*, energy transmission coefficient, *ƞ_t_*, and filling thickness is depicted in Figure 15. The *ƞ_r_* and *ƞ_t_* of jointed rock samples with filling thicknesses of 2.40 mm, 4.91 mm, 7.56 mm, 10.73 mm, and 12.15 mm are 39.12%, 66.05%, 62.13%, 78.56%, 91.23% and 50.22%, 17.08%, 16.35%, 7.22%, 2.50%, respectively. As the filling thickness increases, the energy reflection coefficient shows a linearly increasing trend, while the energy transmission coefficient exhibits a linearly decreasing trend.

#### 5.3.2. The Relationship Between the Energy Dissipation Ratio and Filling Thickness

**Figure 16:**
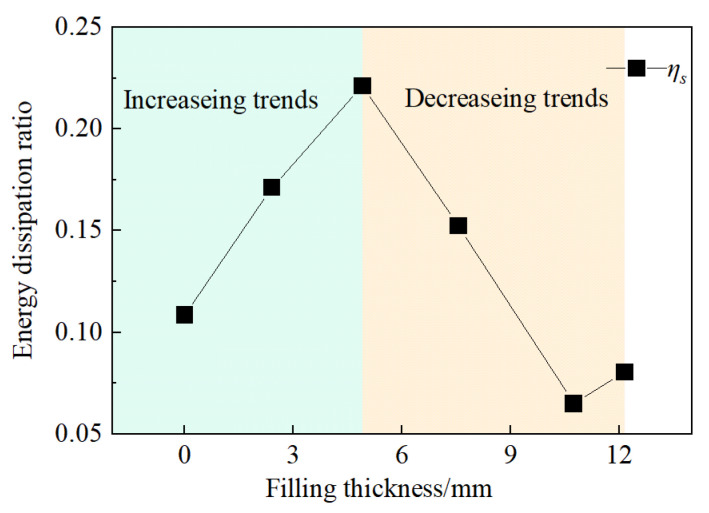
The relationship between energy dissipation ratio and filling thickness.

If we neglect the small fraction of energy loss that occurs during the propagation of stress waves, the majority of the absorbed energy is primarily used for the damage and destruction of the rock samples. Therefore, the magnitude of the energy dissipation ratio can reflect the amount of energy required for the failure of jointed rock samples. The relationship between the energy dissipation ratio, *ƞ_s_*, and filling thickness is shown in Figure 16.

Figure 16 revealed that the energy dissipation coefficient exhibits an initial increase followed by a decrease with an increase in filling thickness. As the filling thickness increases from 0 mm to 4.91 mm, the energy absorption rate of the samples begins to increase due to the ductility and lower strength of the filling material. From 4.91 mm to 12.15 mm, the wave impedance of the grouted joints is lower than that of the hard rock material on both sides, leading to the reflection of most energy back into the incident bar. Consequently, the energy transmitted to the transmission bar and absorbed by the sample is significantly reduced, resulting in a sharp decrease in the energy absorption rate. The fitted curve indicates the existence of a critical joint thickness (approximately 4.91 mm) at which the energy dissipation coefficient is maximized. Filling joints within this thickness range can significantly affect the degree of damage and destruction of the rock samples, increasing the energy required for sample failure.

## 6. Discussion

In blasting projects involving granite with filled joints, attention should be paid to the thickness of the filled joints in the rock mass, especially when the filling thickness reaches around 7 mm, as this may significantly affect the deformation characteristics and energy dissipation patterns of the rock mass. This paper’s findings can guide blasting operations in filled jointed granite for slope and tunnel engineering, improving blasting efficiency and ensuring effective protection.

## 7. Conclusions

By analyzing dynamic impact test data on unfilled jointed granite and granite samples with five different filling thicknesses, along with high-speed camera footage of sample failures during the impact process, we were able to determine the dynamic properties and energy evolution patterns of jointed granite with varying filling thicknesses. The main conclusions are as follows:

The thickness of the filling has a significant impact on the stress–strain curves of jointed rock samples. Compared to unfilled jointed rock samples, those with filled joints display a distinct “stress bimodal” phenomenon. As the thickness of the filled joints increases, the degree of “strain rebound” decreases, resulting in a progressive reduction in the load-bearing capacity of the rock samples during the post-peak unloading phase. Through the camera, we can observe that the primary failure mode of jointed granite is tensile failure. The area of the rock mass adjacent to the incident bar, the lower section of the rock mass, and the gypsum filling are particularly vulnerable to damage. The degree of rock mass damage is minimal when the filled joint thickness is 2.40 mm, and it is most severe when the filled joint thickness is 7.56 mm. When the filled joint thickness exceeds 7.56 mm, stress waves have difficulty penetrating the filled joints. Furthermore, experimental data indicate that jointed rock samples’ dynamic compressive strength and peak strain are correlated with the filling thickness. As the filling thickness increases, the dynamic compressive strength decreases, which indicates that the jointed rock mass becomes less capable of resisting impact loads. The peak strain shows a pattern of first increasing and then decreasing with the increase in filling thickness, reaching a critical joint thickness of approximately 7.56 mm, where the deformation of the rock samples is maximized. With the increase in filling thickness, the reflected energy increases, the transmitted energy decreases, and the energy dissipation ratio exhibits a trend of initially increasing and then decreasing.

## Figures and Tables

**Table 1:** Physical and mechanical parameters of rock and filling materials.

Materials	Mix Ratio	Density/(kg·m^3^)	Uniaxial Tensile Strength/MPa	Elastic Modulus/GPa	Poisson’s Ratio	Wave Velocity/(m/s)
Granite	-	2586	121.56	28.47	0.22	5357
Gypsum filling	Water:Gypsum = 3:2	1800	12.18	0.35	0.25	2485

**Table 2:** Basic physical parameters of rock samples.

Type of Rock Samples	Diameter/mm	Length/mm	Filling Thickness/mm	Volume/×10^−3^ m^3^	Quality/g
nfilled jointed rock samples	50	41.08 41.10 41.18	- - -	80.66 80.70 80.86	200.57 200.35 200.78
Filled jointed rock samples	50	44.40 44.56 44.60 46.10 46.18 46.20 49.10 49.15 49.22 51.50 52.00 52.30 53.00 53.00 53.30	2.40 2.40 2.40 4.82 4.91 5.00 7.44 7.56 7.60 10.50 10.73 10.80 12.00 12.10 12.15	87.18 87.49 87.57 90.52 90.67 90.71 96.41 96.50 96.64 101.12 102.10 102.69 104.07 104.06 104.65	215.50 215.58 215.60 215.59 216.08 216.00 223.52 223.87 224.27 228.72 228.97 229.68 230.71 230.74 230.82

## Data Availability

The original contributions presented in this study are included in the article. Further inquiries can be directed to the corresponding authors.
